# *Letting go with the flow:* directional abscission of dandelion seeds

**DOI:** 10.1098/rsif.2025.0227

**Published:** 2025-09-10

**Authors:** Jena Shields, Fiorella Ramirez-Esquivel, Yukun Sun, Aspen Shih, Sridhar Ravi, Chris Roh

**Affiliations:** ^1^ Applied and Engineering Physics, Cornell University, Ithaca, NY, USA; ^2^ School of Engineering Technology, University of New South Wales, Canberra, ACT, Australia; ^3^ Research School of Biology, Australian National University, Canberra, Australian Capital Territory, Australia; ^4^ Department of Biological and Environmental Engineering, Cornell University, Ithaca, NY, USA

**Keywords:** dandelion, seed abscission, seed dispersal, anemochory, Asteraceae

## Abstract

Seed dispersal through wind was historically considered a random process; however, plants can influence their dispersal through non-random seed detachment or abscission. Dandelion seeds facing the wind tend to abscise before those facing downwind, yet the mechanism that supports this has remained unclear. We measured the force needed for abscission in different directions and performed imaging of the detachment process. This revealed an asymmetry in the seed attachment morphology, which results in massive differences in the abscission force needed relative to the direction. We developed a mechanistic model to explain this directional bias and identified morphological factors that determine the properties of seed abscission. This discovery highlights plant adaptations that shape the seed dispersal profile to enhance reproductive success and can be used to improve population dynamic models of wind-dispersed plants.

## 1. Introduction

Seed dispersal plays a key role in the evolutionary fitness of plant reproduction. Dispersing offspring away from the parent is important because it reduces competition between kin and allows new territories to be colonized [1]. Wind-dispersed seeds develop aerodynamic structures that interact with the air to stay afloat longer, allowing long-range propagation, which furthers these dispersal aims [2–4].

Wind dispersal (anemochory) was historically seen as a passive process, where only environmental conditions determine dispersal. However, recent research has increasingly acknowledged that parent plants can play an active role in seed dispersal to promote dispersal under favourable environmental conditions [3,5–7]. Plants achieve this through non-random seed abscission, where the probability of abscission depends on environmental conditions [3,4,8]. The conditions in which the seed abscises become the initial conditions of the seed’s dispersal. Thus, non-random abscission can alter the dispersal profile and therefore the plant’s population distribution.

The common dandelion, *Taraxacum officinale*, has been shown to exhibit such non-random seed abscission [7–9]. Its seed dispersal strategies are at least partially responsible for its nearly worldwide distribution and evolutionary success [10]. A quintessential example of an anemochorous plant, dandelion seeds respond directly to wind conditions or indirectly to other environmental conditions by changing the aerodynamic quality of the seed. The dandelion seed breaks off when a critical wind speed is reached, so that flight is initiated under long-distance favouring strong winds [11]. In contrast, under unfavourable rainy conditions, the dandelion seed closes its pappus (fluffy white hairs) to limit abscission by reducing aerodynamic drag [8].

Research on seed dispersal has increasingly considered the effect of wind magnitude on seed abscission, and this has been found to be important for accurate dispersal modelling [2,8,9,11–22]. However, the role of wind direction has not been included in these models even though several studies report that dandelion seed abscission is wind direction sensitive. Dandelion seed abscission starts on the windward versus the leeward side of the seed head [5,9]. Moreover, updraughts and horizontal winds abscise seeds more readily than downdraughts. This is thought to disperse seeds farther away from the parent plants by lifting the seeds out of the slow-moving flow near the ground [5,6,14,20,23]. However, a morphological model that explains directionally sensitive abscission has not yet been proposed. This leads to an incomplete picture of the dynamics of dispersal, which limits accurate ecological modelling of seed dispersal under a growingly unpredictable climate.

Here, we investigate the morphological origin of directional sensitivity in the dandelion seed abscission. We show that the abscission force can vary over an order of magnitude depending on the direction the force is applied. Through a quantitative mechanistic model, we identify morphological features, which appear to give rise to the seed’s asymmetric response to wind direction.

## 2. Methods

### 2.1. Scanning electron microscopy imaging

Scanning electron microscopy (SEM) imaging was carried out in Canberra, Australia (ASTL) and Ithaca, New York (USA). Dandelion seed heads (*Taraxacum officinale*) were dried and cut into regions of interest using a razor blade (USA) or scalpel blade (ASTL). ASTL segments were dehydrated in an ethanol series (50, 70, 80, 90, 100 and 100%), samples were held at each concentration for 10 min, then held overnight in the final 100% solution. Samples were then removed and placed on tissue paper to draw excess moisture and allowed to air dry. USA samples were air dried then cut. Dandelion fragments were mounted onto aluminium stubs using adhesive carbon tabs and silver paint. Final drying was performed in a 40°C oven overnight (ASTL). The samples were then sputter coated (Au, 2 min, 20 mA) in ASTL using an EMITech K550X (Quorum Technologies Ltd., Lewes, UK) and (Au-Pd, 2 min, 30 mA) in the USA using a Denton Desk V sputter coater (Denton Vacuum, Moorestown, NY, USA). Samples were imaged using a Zeiss UltraPlus FESEM (Carl Zeiss GmbH, Oberkochen, Germany) under an accelerating voltage of 3 kV (ASTL) and using a Tescan Mira3 FESEM (TESCAN, Brno, Czech Republic) with an accelerating voltage of 1 kV (USA). Measurements were carried out using ImageJ 1.53t (Rasband, National Institutes of Health, USA).

### 2.2. Confocal laser microscopy

Dandelion achenes were embedded in 6% (w/v) agarose (SeaKem, Lonza Bioscience, Walkersville, MD) in PBS gel. After solidification, a vibrating microtome (Integraslice 7550 PSDS, Campden Instruments, Leicester, UK) was used to create 200 µm nominal thickness sections (100 Hz, 1.0 mm amplitude, 0.26 mm s^−1^ advance speed). Sections were placed facing upward on microscope slides. Equal volumes of 10% (w/v) potassium hydroxide solution and 1 mg ml^−1^ Calcofluor White (no.18909, Sigma Aldrich, St. Louis, MO) were pipetted onto the sections, then covered with a no.1.5 22 × 40 mm coverslip (no.72204-03, Electron Microscopy Sciences, Hatfield, PA) and sealed with nail polish. The samples were left for over 10 minutes before imaging.

Slides were imaged with a scanning laser confocal microscope (LSM 880, Carl Zeiss GmbH) with 405 and 488 nm excitation through a 63 x/1.2 NA C-Apochromat W Korr objective lens (Carl Zeiss GmbH). Fluorescence of the Calcofluor White dye was induced by 405 nm excitation and imaged with a PMT detecting the 410−495 nm emission band, and sequential 488 nm excitation induced autofluorescence of the tissue, which was imaged with a PMT detecting the 495−630 nm emission band. Sequential acquisition of 512 × 512 pixel images was performed at 0.134 µm pixel edge length.

### 2.3. Micro-computed tomography imaging

Micro-computed tomography (CT) imaging was performed at the Cornell Institute of Biotechnology’s Imaging Facility. Dandelion seed heads were put in 50 ml Tygon tubes and imaged using a microCT device (Skyscan 1276, Bruker, Billerica, MA, USA). The CT scanned the dandelion seed head every 10 µm.

### 2.4. Seed characteristics measurements

**Figure 1.**
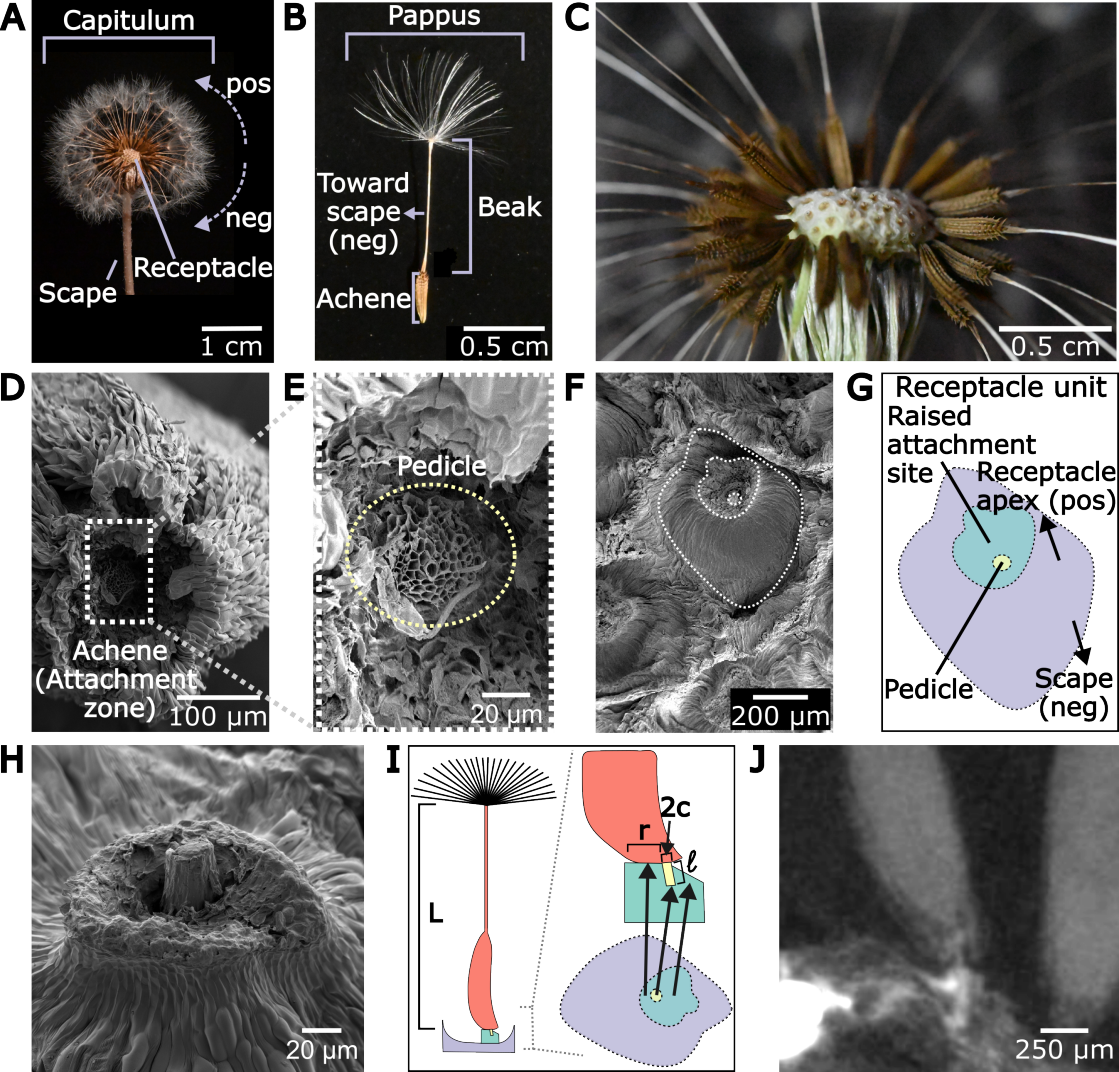
The structure and morphology of the dandelion. (A) The dandelion capitulum is supported by the scape and comprises the receptacle and the attached seeds. Pulling seeds towards the capitulum apex is considered the positive direction (pos) and pulling seeds towards the scape is considered the negative direction (neg). (B) Each seed unit consists of a pappus, beak and achene. (C) Close-up view of seeds attached to the receptacle. Each ‘bump’ attaches to one seed. (D,E) The base of the achene connects to the receptacle through the porous pedicle, which extends into the base of the achene. (F) The structure of the receptacle. A singular receptacle unit is outlined in white. (G) The receptacle unit has a raised attachment site (blue) from which the pedicle extends (yellow). The arrows indicate the directions towards the scape and the receptacle apex. (H) Angled view of one raised attachment site and pedicle. The attachment site is seen to be higher behind the pedicle and lower in front of the pedicle. (I) Schematic of the dandelion seed and its connection to the attachment site. Side view and top view of the receptacle unit are seen on the right. The pedicle is in yellow, with radius c and length ℓ, achene/beak in red with length L and attachment site in blue. The attachment site is raised on one side and not the other. The raised section has a length of r. (J) CT image of a dandelion seed attached to a receptacle unit. The seed is in contact with the attachment site on the left and not on the right.

**Figure 2.**
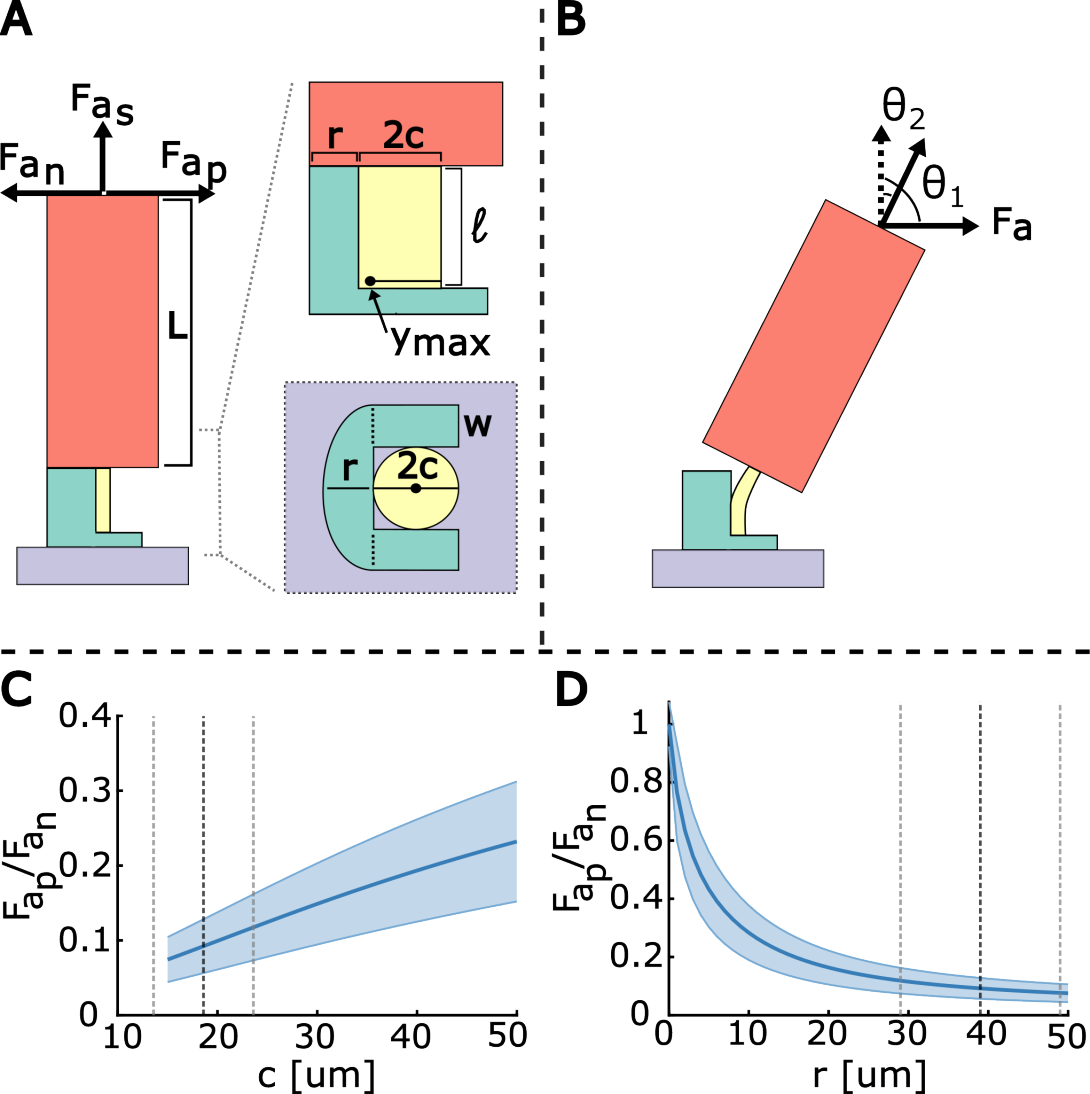
Theoretical model of dandelion seed abscission. (A) The dandelion seed model, and its connection to the attachment site, based on the schematic shown in figure 1I. Side view and top view of receptacle unit are seen on the right. The pedicle is in yellow, achene/beak in red and attachment site in blue. The raised portion of the attachment site is modelled as a horseshoe. The model neutral axis $y_{max}$ lies within the pedicle. Forces are applied at the top of the achene model. (B) A force is applied to the top to the seed at an angle $\theta_{1}$ to the seed’s original axis (dotted line). The seed abscises at an angle $\theta_{2}$ to the original axis. (C,D) The ratio $F_{a_{p}}/F_{a_{n}}$ varies as the value of *c* and *r* change, respectively, at $\beta^{*}=25.9$. The shaded area shows the propagated error of the force ratios, and dotted lines are average values and standard deviations of the parameters c and r, respectively.

Seed length measurements (L) were taken using a calliper from the centre of the pappus to the tip of the achene (*n* = 304, from 20 seed heads). The attachment site radius (r) (*n* = 104), defined as the distance from the pedicle to the edge of the attachment zone on the side towards the scape, the attachment site width (w) (*n* = 228), defined as the thickest distance from the pedicle in the perpendicular direction of *r*, and pedicle radius (c) (*n* = 115) were measured from SEM imaging of five different seed heads using ImageJ 1.53t (Rasband, National Institutes of Health, USA). These four parameters are shown in figures 1I and 2A. Pedicle porosity (*k*) (*n* = 10) was measured using imageJ from one SEM image to measure the size of individual vascular tubes in the vascular bundle (figure 1E, electronic supplementary material, figure S1). The area of the entire vascular tube was measured, and then the area of the hollow centre was measured. The fraction of the difference between the two areas over the total area was taken as the porosity. The average values for each are shown in table 1 with bootstrapped error in subscript and superscript and standard deviation reported afterward.

**Table 1.** Morphological parameters of dandelion seed. The mean value, bootstrapped error in subscript and superscript and standard deviation for seed length, base width, base radius, pedicle radius and pedicle porosity are given along with the number of measurements in parentheses.

parameter	value
seed length (*L*)	$12.4_{-0.1}^{+0.1}\pm 1$ mm (*n* = 304)
base width (*w*)	$64_{-2}^{+2}\pm 16$ µm (*n* = 228)
base radius (*r*)	$39_{-2}^{+2}\pm 10$ µm (*n* = 104)
pedicle radius (*c*)	$18.6_{-0.9}^{+1.0}\pm 5$ µm (*n* = 115)
pedicle porosity (*k*)	$0.33_{-0.05}^{+0.05}\pm 0.1$ (*n* = 10)

### 2.5. Seed in the wind video

The video was taken using a Chronos CR21-HD high-speed camera (Kron Technologies, Burnaby, BC, Canada) with a LAOWA FF 100 mm F2.8 CA-Dreamer Macro 2× lens (Venus Optics, Hong Kong) at 300 fps. The wind was generated anthropogenically on demand, uncharacterized. Wind speed increased from 0 m s^−1^ until seeds began to disperse from the seed head.

### 2.6. Seed abscission videos

Videos of seed abscission were taken using an Olympus SZX12 stereoscope (Olympus America Inc., Center Valley, PA, USA) fitted with an Olympus EP50 camera (Olympus America Inc.) or with a LEICA MZ16 FA microscrope (Leica Microsystems, Wetzlar, Germany) with a Nikon Z5 camera (Nikon Corp., Tokyo, Japan). Dandelion stems were held in place with wax, double-sided tape and superglue, on a manual tilting micro-stage. This micro-stage was tilted and rotated until the desired seed was in the field of view of the stereoscope. Then the dandelion seeds were pulled off in different directions using tweezers pulling on the pappus. Videos were taken at 63× to 115× magnification.

### 2.7. Force measurements

**Figure 3.**
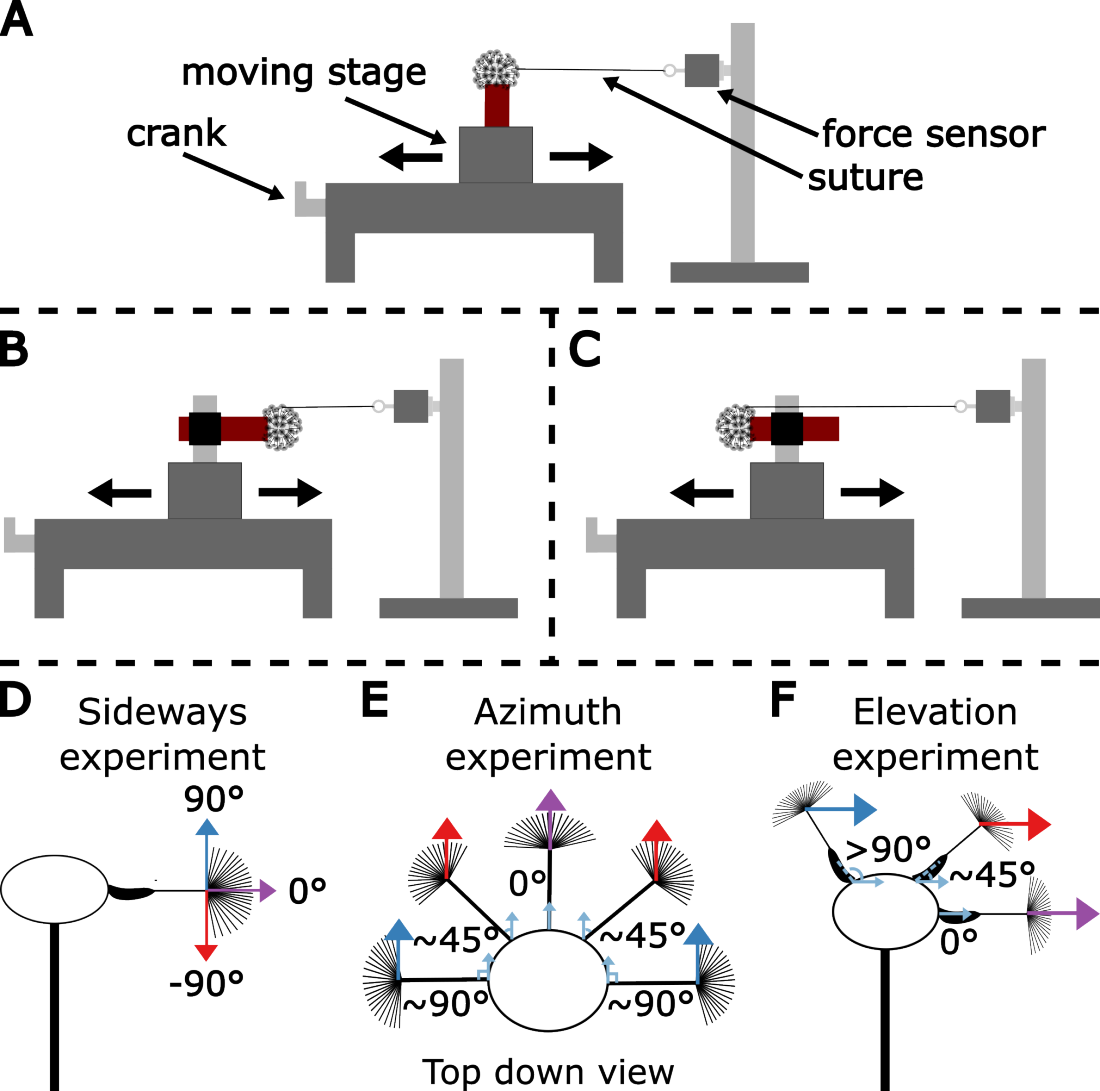
Experimental set-up of force measurement experiments. (A–C) The dandelion seed head was attached to a micro-stage through a stem holder (seen in red). An individual seed was attached to the force sensor through a suture glued to the centre of a seed’s pappus. The whole seed head was moved away manually by the micro-stage’s crank until abscission. The seed head was attached in various positions to vary to force angle and orientation. (D) The sideways force experiment pulled seeds off in three different directions, in the positive direction ($90^{\circ }$), straight out from the seed head ($0^{\circ }$) and in the negative direction ($-90^{\circ }$). (E) The azimuth experiment varied the angle seeds were pulled off in the azimuth plane between 0° and 90°. (F) The elevation experiment varied the angle seeds were pulled off in the elevation plane between 0° and 135°.

**Figure 4.**
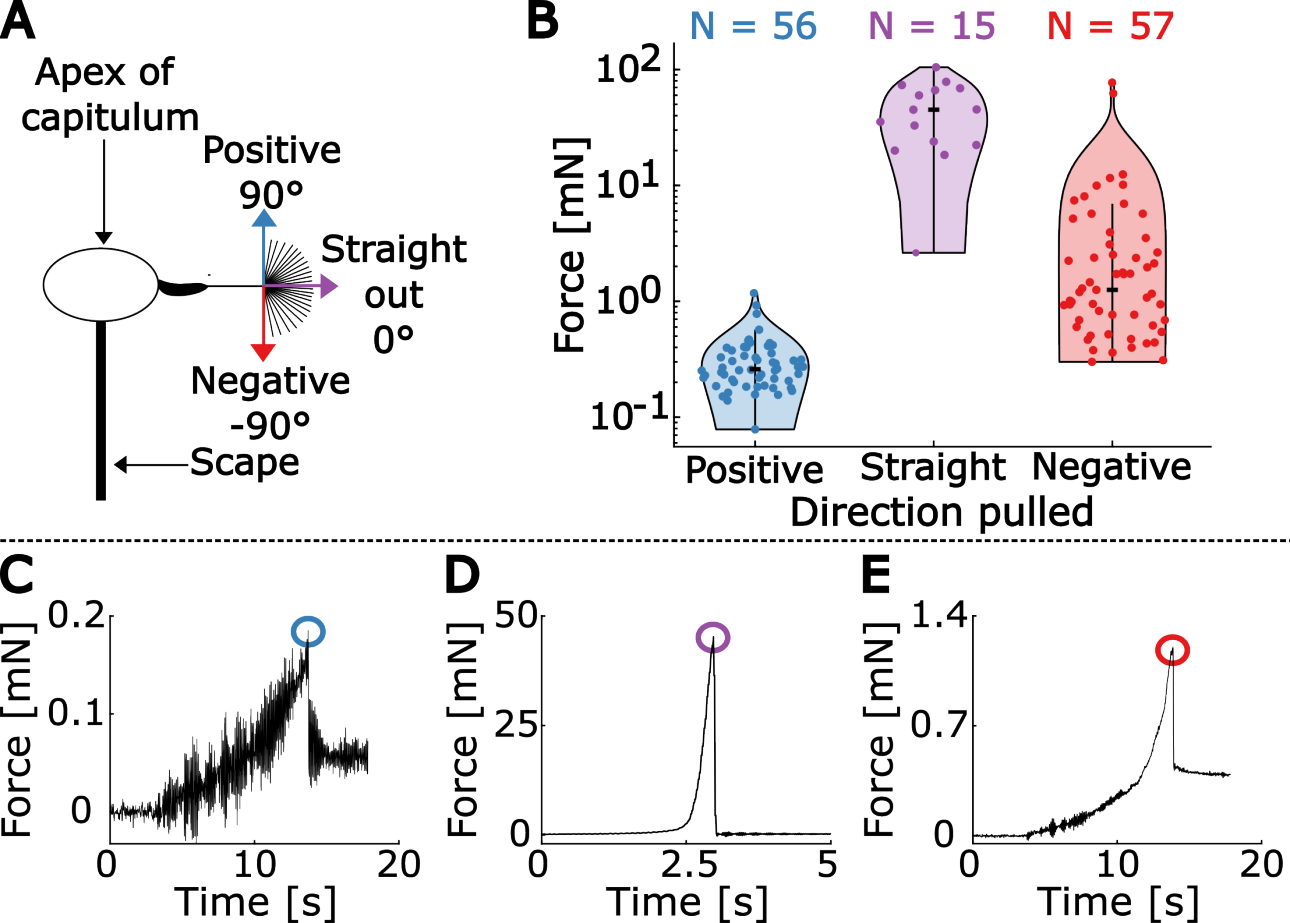
Results of sideways abscission force measurement experiments. (A) Seeds are pulled in three directions: straight out from the receptacle ($0^{\circ }$), positively towards the apex of the receptacle ($90^{\circ }$) or negatively towards the scape ($-90^{\circ }$). (B) Force measurement results from the experiment. Force (mN) is plotted in the log scale, overlayed with violin plots [24]. Median is marked by a thick black dash. (C–E) Sample force measurement time traces of pulling towards the positive, straight out and negative directions, respectively, with the measured abscission force circled. The residual forces after abscission are due to some parts of the seed still making contact with the seed head.

Force measurements were taken using a S-beam load cell (LSB200, FUTEK, Irvine, CA) at 100 Hz sampling rate with a maximum force range of 10 g. An eye bolt attached to one end of a strand of surgical suture was connected to the FUTEK sensor. Dandelion stems (*Taraxacum officinale*) were cut to approximately an inch long, a metal pin was inserted through the hollow stem into the seed head to help prevent twisting, and the stem/pin unit was inserted into a three-dimensional printed stem holder filled with water to prevent wilting. The bottom of the seed head was attached to the top platform of the stem holder using cyanoacrylate glue. The stem holder was fastened to a micro-stage. A single seed was attached to the force sensor by gluing (Loctite, UV Resin) the centre of the pappus to the free end of the suture, to mimic a wind acting upon the pappus (figure 3A–C). At this point, the height of the force sensor was varied to match the height of the seed so that the suture was horizontal. The angle between the suture and seed was measured by eye with a protractor as a measurement of the force angle. After, the micro-stage was moved manually at a sufficiently slow speed to approximate static loading to pull the entire seed head away from the force sensor until the seed abscised. The force measured at this point was considered the abscission force (figure 4C–E).

Three sets of experiments were conducted: an azimuth experiment (*n* = 75) (figure 3E), an elevation experiment (*n* = 90) (figure 3F) and a sideways experiment (*n* = 128) (figure 3D). For the azimuth experiment, the stem holder was held vertically (figure 3A), and seeds were pulled at varying azimuth (horizontal) angles (figure 3E). For the elevation experiment, the stem holder was held vertically (figure 3A) and seeds were pulled at varying elevation degrees (figure 3F). For the sideways experiment, the stem holder was held sideways with either the top of the capitulum or the stem facing the force sensor (figure 3B,C). Seeds were pulled either straight out of the seed head (0°), or at 90° angles either towards the scape (negative) or towards the apex of the capitulum (positive) (figure 3D).

### 2.8. Data analysis

Since the force data are not normally distributed, the medians and interquartile ranges are reported for each population (table 2). To determine the significance of the medians of different populations, the Wilcoxon rank sum test was used. Error was estimated by bootstrapping the data 1000 times and taking the 25th and 975th values.

**Table 2.** Summary of results of force measurement experiments. For each angle and experiment bin, the medians [mN] of the data populations are reported along with the bootstrapped error seen in parentheses and the interquartile range (Q1–Q3).

experiment	angle	median force (mN)	interquartile range (mN)
sideways	straight out $\left(0^{\circ }\right)$	$45_{-21}^{+21}$	23–68
sideways	negative $\left(-90^{\circ }\right)$	$1.3_{-0.3}^{+0.7}$	0.7–3.2
sideways	positive $\left(+90^{\circ }\right)$	$0.26_{-0.03}^{+0.05}$	0.20–0.37
azimuth	$0^{\circ }$	$54_{-23}^{+19}$	28–83
azimuth	$∼45^{\circ }$	$2.3_{-1.2}^{+2.0}$	1.0–5.1
azimuth	$∼90^{\circ }$	$0.71_{-0.23}^{+0.23}$	0.47–1.08
elevation	$0^{\circ }$	$47_{-16}^{+10}$	28–75
elevation	$0^{\circ }<\theta <90^{\circ }$	$2.0_{-1.0}^{+2.0}$	0.8–7.1
elevation	$\theta >90^{\circ }$	$0.30_{-0.03}^{+0.13}$	0.25–0.44

## 3. Results

### 3.1. Dandelion morphology

The dandelion seed head, or capitulum, is attached to the parent plant through a hollow stem, the scape (figure 1A). Each capitulum has around 100−300 seed units [10]. The seed unit is composed of a fruit, or achene, which attaches to the pappus through a thin beak (figure 1B). The pappus consists of radially extending fibres, which are responsible for seed flight during dispersal [8,25,26]. The achenes connect to the capitulum at the receptacle through a thin, porous, vascular bundle, the pedicle (figure 1D–G, electronic supplementary material, figure S1).

### 3.2. Abscission force measurements

To assess the directional sensitivity of abscission, we directly measured the force of abscission by pulling the achenes in different directions. In the sideways experiment, we pulled seeds: outward normal to the receptacle (0°), negatively towards the scape at −90°, or positively towards the apex of the receptacle at +90° (figure 4A). The seed was pulled with a string glued to the centre of the pappus at the top of the beak (figure 3B,C). The median force for each regime is: $45_{-21}^{+21}$, $1.3_{-0.3}^{+0.7}$ and $0.26_{-0.03}^{+0.05}$ mN, respectively, with the bootstrapped error shown as superscript and subscript (see §2 for details). Pulling seeds at an angle (±90°) compared with straight out (0°), decreases the force needed for abscission by orders of magnitude (table 2, figure 4B). Additionally, pulling seeds positively (+90°) requires an order of magnitude less force than pulling seeds negatively (−90°). The differences in force required for pulling straight out versus positively, pulling straight out versus negatively and pulling positively versus negatively are all significant (Wilcoxon rank sum test *p*-values: 3 × 10^−^⁹, 6 × 10^−^⁸ and 8 × 10^−^¹⁷, respectively).

To test the effect of different angles, two additional force measurement experiments were conducted, which varied the azimuth and elevation angle of the applied force (figure 3E,F). These experiments show that as the angle increases, the force needed for abscission decreases in both the elevation and azimuthal planes (electronic supplementary material, figure S2, table 2).

In the azimuth force measurement experiment, the seeds were pulled off the seed head at varying azimuth (horizontal) angles (figure 3E). These angles were approximately $0^{\circ }$, $45^{\circ }$ and $90^{\circ }$ between the seed and the force sensor. The results, summarized by table 2 and electronic supplementary material, figure S2B, show that increasing the angle decreases the force needed for abscission by orders of magnitude. The difference in force between 0° and 45°, 0° and 90° and 45° and 90° are all significant (Wilcoxon rank sum test *p*-values: 2 × 10^−^⁹, 2 × 10^−9^ and 7 × 10^−4^, respectively).

In the elevation force measurement experiment, the seeds were pulled off at different elevation (vertical) angles (figure 3F). These angles were binned into three categories: 0°, acute degrees and obtuse degrees. The results, summarized in table 2 and shown in electronic supplementary material, figure S2D, show that increasing the elevation angle seeds are pulled at decreases the force needed for abscission. There is an order of magnitude difference between pulling a seed at angles greater than 90° compared with 0° or acute degrees. Therefore, when seeds are pulled towards the capitulum apex (positive) versus towards the scape (negative), there is an order of magnitude difference in force needed for abscission. The difference in force between 0° and acute degrees, 0° and obtuse degrees and acute degrees and obtuse degrees is all significant (Wilcoxon rank sum test *p*-values: 2 × 10^−7^, 7 × 10^−8^ and 4 × 10^−5^, respectively).

### 3.3. Dandelion imaging

**Figure 5.**
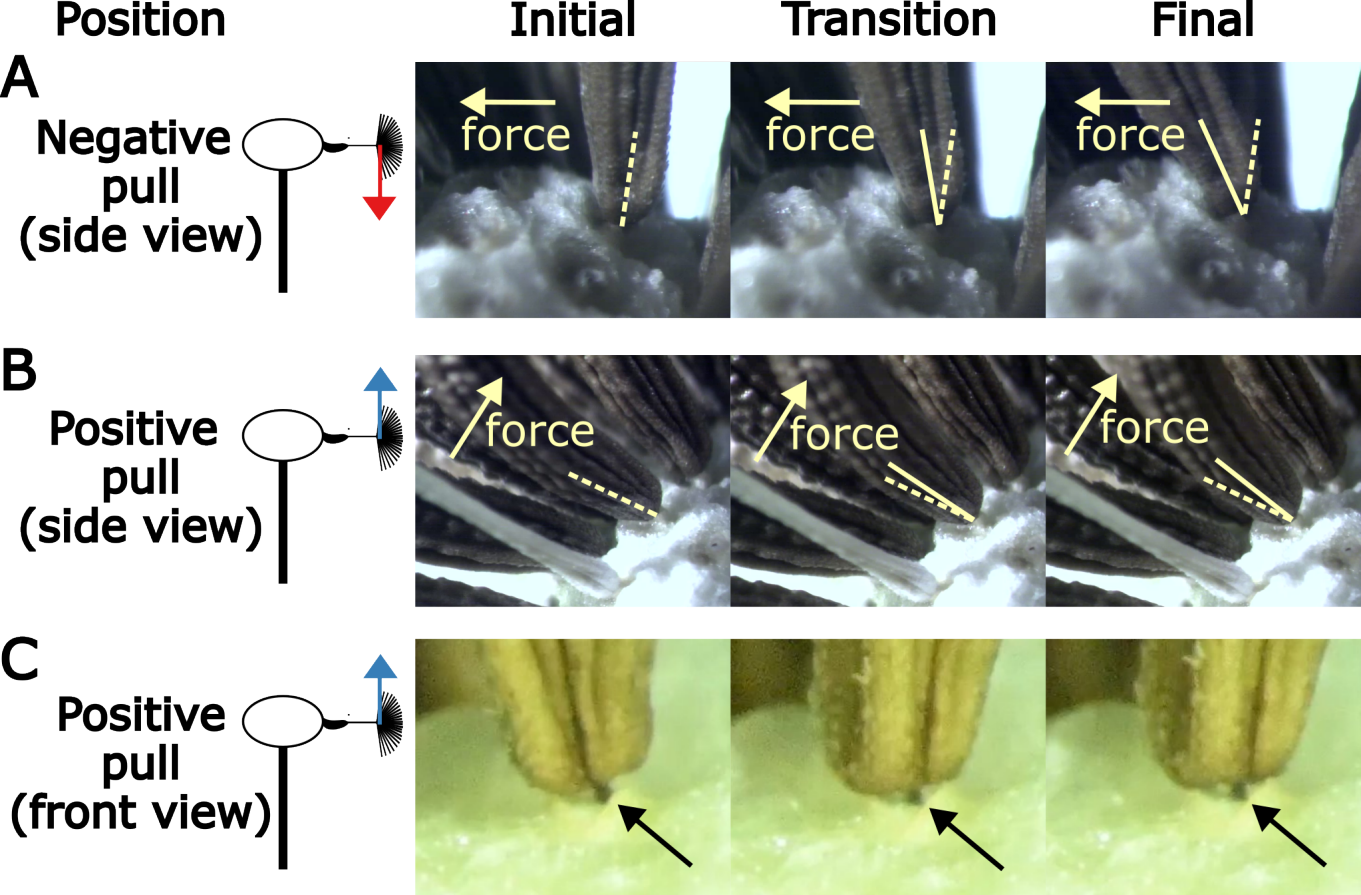
Abscission montage of seeds pulled in different directions. The left column of images shows the initial position of the seed with no force. The middle column shows a transitional position as the seed is being pulled. The right column is the position of the seed right before abscission. (A) Montage of a seed being pulled negatively towards the scape, side view. The seed moves approximately $35^{\circ }$. (B) Montage of a seed being pulled positively towards the apex of the capitulum, side view. The seed moves approximately $15^{\circ }$. (C) Montage of a seed being pulled positively towards the apex of the capitulum, front view. The black arrow points to the pedicle (black tissue), which is visible during the entire abscission process.

To determine whether this asymmetrical abscission behaviour of the achene is reflected in the receptacle morphology, we examined achenes and receptacles under SEM, confocal laser microscopy and through abscission videos (electronic supplementary material, videos S1–S4, figure 5). Figure 1C shows an image of the receptacle with seeds removed. The receptacle consists of individual units, each consisting of roughly circular craters, called the sheath, with a raised attachment site inside. The attachment site is located on the side of the receptacle unit closer to the receptacle apex (figure 1F,G). Each receptacle unit attaches to an achene through the pedicle that extends out of the attachment site.

The raised attachment site is conspicuously asymmetric and semicircular with a small protrusion that almost resembles a mushroom or bell shape (figure 1F–H). When a seed is attached to the receptacle, the outward curved region of the achene is in contact with the wide, semicircular, raised portion of the attachment site, resembling a horseshoe and behind the pedicle (figure 1H), while the achene does not touch the small protrusion of the attachment site located towards the apex of the capitulum, in front of the pedicle (figure 1H). Figure 1 shows a CT scan of the attachment of a seed to the receptacle, the seed is in contact with the attachment site on the left (negative direction) and not on the right (positive direction). These observations inform our schematic (figure 1I), where the achene is supported on one side only.

The pedicle is seen in greater detail through SEM imaging (figure 1D,E) and confocal laser microscopy (electronic supplementary material, figure S1). These images show that the pedicle is porous.

The parameters c, r and w, do have considerable natural variation (table 1), but do not show general trends with distance from the centre of the capitulum (electronic supplementary material, figure S3A). The main variation from this is that the outermost edge of receptacle units (which mostly have seeds pointing towards the ground) change shape (electronic supplementary material, figure S3B). This is primarily observed through the increased width of the attachment site. Since this has only been seen to occur in the outermost edge cases, we ignore this shape change in our model and analysis.

Videos of the abscission process show that when a force is applied to the seed in the negative direction (towards the scape), the receptacle deforms due to compression before the seed breaks off (electronic supplementary material, video S1, figure 5A). The contact and deformation of the receptacle suggest that it provides a resistive force to the applied force.

In contrast, when a force is applied to the seed in the positive direction (towards the receptacle apex), the seed abscises after a small rotation (electronic supplementary material, video S2, figure 5B). There is limited contact between the seed and the receptacle in the positive direction, evidenced by the usually visible pedicle through the gap between the receptacle and the achene (electronic supplementary material, video S3, figure 5C). The absence of contact suggests that the receptacle base only provides a resistive force on the sides of the achene.

Additionally, when seeds are pulled straight out from the receptacle, the receptacle often deforms outward before the seed breaks off (electronic supplementary material, video S4).

### 3.4. Mechanistic model

The dandelion beak and achene are modelled as a large cylinder (achene model) of length L and radius r+c (in red) connected to the attachment site base (in blue) by a small cylinder (pedicle model) with length ℓ and radius c (in yellow). The base is asymmetric and supports the bottom of the achene model in a horseshoe-like shape with no support on the positive side (figure 2A). The sides of the base are rectangles of size 2c×w and the negative side is a semi-ellipse with axes r and c+w (figure 2A).

A force $F_{a}$, is applied at an angle $\theta_{1}$ to the vertical normal. The seed abscises at $|\theta_{2}|\leq \left|\theta_{1}\right|$, where $\theta_{2}$ is defined as the angle of the model achene axis to the vertical (figure 2B). When the pedicle is pulled to the side, part of the horseshoe attachment site is put into compression. This section of the base and the pedicle bend as one unit.

$F_{a_{s}}$ is the abscission force when $\theta_{1}=0$ (electronic supplementary material, figure S4A). $F_{a_{n}}$ is the abscission force when pulling the model achene negatively towards the side with the raised base (electronic supplementary material, figure S4B). $F_{a_{p}}$ is the abscission force when the model achene is pulled positively towards the other side (electronic supplementary material, figure S4C).

When $\theta_{1}\neq 0$, the applied force $F_{a}$ exerts both a tension and a bending (torque) component on the pedicle. The tension component is the force parallel to the pedicle. The bending moment comes from the torque balance between the moment of $F_{a}$ and the bending moment within the pedicle. Both components contribute to the total stress exerted on the pedicle. When this total stress reaches the pedicle’s breaking stress, the seed abscises. Stresses from tension, $\sigma_{t}$, and bending, $\sigma_{b}$, are defined as [27]


(3.1) $$\sigma_{t}=\frac{F_{t}}{A},\sigma_{b}=\frac{My_{\max}}{I},$$

where $F_{t}$ is the applied tension component, A is the cross-sectional area, M is the bending moment, $y_{max}$ is the distance between the neutral axis and the edge in tension (figure 2A) and I is the second moment of area. Using the stresses produced by a force applied at $\theta_{1}\neq 0$ compared with $\theta_{1}=0$, we get the relationship


(3.2) $$\frac{F_{a}}{F_{a_{s}}}=\frac{1}{\cos(|\theta_{1}|-\frac{|\theta_{2}|}{2})+\frac{Ay_{max}}{I}p},$$

where $y_{\text{max}}$ is the distance from the neutral axis of the pedicle-base bending unit to the outer edge of the pedicle (figure 2A), I is the second moment of area of the pedicle-base bending unit, p is the moment arm of bending between $F_{a}$ to the base of the pedicle and $A=k\pi c^{2}$ is the cross-sectional area of the model pedicle. The value k defines the porosity of the pedicle. The value of p depends on L, ℓ, $\theta_{1}$ and $\theta_{2}$ (see electronic supplementary material for details).

Due to the asymmetry of the horseshoe attachment site (figure 2A), the values of $y_{\max}$ and I differ in the positive and negative directions. The exact calculation of these values depends on the elastic moduli of the base ($E_{b}$) and pedicle ($E_{p}$), which are not measured. We work under the assumption that $\beta =E_{p}/E_{b}>1$ due to the pedicle being brittle and stiff while the base is deformable. We only consider the model valid when $y_{\max}$ is within the pedicle: $2c\geq y_{\max}\geq c$ (figure 2A). Additionally, when $\frac{L}{\ell }\geq 20$, $\frac{\theta_{1}}{\theta_{2}}\geq 3/2$ and $|\theta_{1}|-|\theta_{2}|\geq \pi /4$, we can disregard the contribution to stress from the tension force and approximate $p\approx L\;\sin(|\theta_{1}|-|\theta_{2}|)$. This approximates the force ratio as


(3.3) $$\frac{F_{a}}{F_{a_{s}}}=\frac{I}{k\pi c^{2}y_{\max}L\sin(|\theta_{1}|-|\theta_{2}|)}.$$

We now have the relationships between the three abscission forces, $F_{a_{s}}$, $F_{a_{n}}$ and $F_{a_{p}}$ (figure 2A), expressed through the parameters β, r, w, c, L, k, $\theta_{1}$ and $\theta_{2}$ (see electronic supplementary material for full derivation).

The abscission videos show that pulling the achene negatively leads to $\theta_{2}\approx 35^{\circ }$ and pulling positively leads to $\theta_{2}\approx 15^{\circ }$ (figure 5, electronic supplementary material, videos S1, S2). Using these values of $\theta_{2}$, $\theta_{1}=90^{\circ }$ and equation (3.3), the force ratios can be calculated for various values of β. When compared with the experimental values, we find the best fit for the model at $\beta^{*}$= 25.9 (electronic supplementary material, figure S4H).

The experimental results and the theoretical values at $\beta^{*}$ are as follows. $F_{a_{p}}/F_{a_{s}}$ is experimentally 0.005 (+0.002 −0.002) and theoretically 0.0026±0.0009. $F_{a_{n}}/F_{a_{s}}$ is experimentally 0.03 (+0.02 −0.01) and theoretically 0.028±0.006. Therefore, $F_{a_{p}}/F_{a_{n}}$ is experimentally 0.21 (+0.12 −0.05) and theoretically 0.09±0.04. The experimental values are provided as medians with bootstrapped error in parentheses. The theoretical values are calculated from equation (3.3) with the values in table 1 and error propagated from parameter bootstrapped errors. These results agree by order of magnitude and are summarized in electronic supplementary material, table S1.

We additionally examined the model as the values of r and c changed independently (figure 2C,D). We scale w=1.6×r. As c increases, the total force needed to abscise the seed increases (assuming the breaking stress stays the same). Varying c changes the force threshold for abscission. Additionally, increasing c while keeping r unchanged decreases the effect of the asymmetry between pulling in the positive and negative directions (figure 2C). Varying r most directly affects the force needed to abscise the seed in the negative direction. As r decreases, the asymmetry decreases (figure 2D), and it becomes easier to abscise in the negative direction. Tuning r can vary the difference in the force needed to abscise seeds in the negative and positive direction.

## 4. Discussion

A seed pulled in the negative direction requires an order of magnitude higher force to abscise than when pulled in the positive direction. Another order of magnitude increase in force is required when a seed is pulled straight out (table 2). High magnification imaging revealed a consistent morphological asymmetry in the tissue surrounding the pedicle. We investigated if this asymmetry could be the cause of the directionally dependent variation needed for abscission force by building a mechanistic model. Our model incorporates the highlighted morphology to explain the origin of directional bias in dandelion seed abscission. There is good order-of-magnitude agreement between the model and experimental results (electronic supplementary material, table S1).

The abscission process, which ultimately demands the breakage of the pedicle, shows lack of support from the receptacle tissue in the positive abscising direction (figure 5B,C, electronic supplementary material, videos S2 and S3), suggesting that when the seed is pulled in positive direction (figure 5B), the pedicle experiences pure bending, which concentrates the stress on the outer edge of the pedicle, which initiates a crack. Pulling the seed straight out distributes the stress throughout the pedicle, needing more force to initiate a crack on the edge. This is consistent with previous works, which suggested that applying torque causes abscission [2,13,14,28,29]. When pulled in the negative direction (figure 5A), there is receptacle tissue support (figure 5A, electronic supplementary material, video S1). We posit this leads to some of the applied stress being distributed to the receptacle tissue, which deforms with the pedicle, lowering the amount of stress applied to the pedicle edge.

Previous studies have offered qualitative and quantitative explanations for the directional preference in abscission. The Greene & Quesada [5] study proposed a qualitative description of the preferential abscission observed in salsify (*Tragopogon Dubius*), a relative of the dandelion [5]. The study suggested that when a seed experiences an updraught (equivalent to positive pulling), the receptacle sheath acts as a fulcrum, whereas, in a downdraught (equivalent to negative pulling), no lever effect is present and instead the seed is compressed against the sheath. The same phenomena are not observed in the dandelion seed abscission process (figure 5, electronic supplementary material, videos S1−S3).

In other works [28,29], the abscission process was modelled based on contact friction between the sides of the lower achene and the receptacle unit. However, in our microscope imaging of mature dandelion seeds, the seed rarely makes contact between these two tissues (figure 5, electronic supplementary material, videos S1−S4).

Our proposed asymmetric abscission process can explain why seeds have been qualitatively observed to abscise on the windward side of the seed head before the leeward side [5,9]. Previous studies attributed this phenomenon to seeds on the windward side shielding those downwind. Although this effect certainly exists, the morphological asymmetry also has significant contribution. This is shown by how the abscission force is 4.8 times greater in the negative direction than the positive direction when no wind effects are present (electronic supplementary material, table S1). The morphology alone is therefore sufficient to generate an asymmetric abscission response. Seeds on the windward side of the seed head will have their pappus pushed downwind, mostly in the positive direction. Seeds on the leeward side of the seed head will bend less since the pappus is already downwind of the seed head (electronic supplementary material, figure S5) and will primarily bend in the negative direction. This means that leeward seeds also need a greater force magnitude, or wind speed, to abscise. Assuming that the wind acts primarily upon the seed units through a drag force, $F∼U^{2}$, this indicates that a wind abscising a seed moving in the negative direction needs to be 2.2 x faster than an wind pushing a seed in the positive direction.

The findings presented here are an integral step in understanding the role of the wind as a vector, both in magnitude and direction, in seed abscission. Previous studies have shown that environmental conditions affect the probability of seed abscission, such as the likelihood of abscission decreasing with humidity [7,8,14,30,31], increasing with turbulence [17,30], increasing with seed head age [16,20,23,30] and increasing with wind speed [5,9,12,14,17,20,23,30,31]. Many of these variables have been incorporated into seed dispersal models for more accurate modelling, but wind direction has not. Using our fitted $\beta^{*}(E_{p}/E_{b})$, our model could be incorporated into current ecological seed dispersal models [2,13].

With this theory of the morphological origin of directional sensitivity, we can also model the effects of changing the size and shape of the attachment site on dandelion seed abscission. If the value of ‘r’ (figure 1I) is reduced, we see that the degree of the asymmetry is reduced. In a situation where the directional bias in the abscission process would decrease or increase the fitness of the organism, it would be interesting to see the shift in dandelion population with a different size and shape of its attachment site. For example, perhaps in an environment with a predominant wind direction, the plant would have greater fitness with greater asymmetrical abscission compared with an environment with no preferred wind direction, due to the changes the increased asymmetry would have on the dispersal profile.

The directionally dependent abscission of dandelion seeds, if coupled with an investigation of the flow structure through a dandelion seed head, could lead to a probabilistic model of abscission given the local wind direction and magnitude. However, this proposed model needs additional testing. To do so would require a study that linked individual force measurements to parameter values and measurements of the Young’s moduli of the receptacle and pedicle tissues. Such measurements would enable further validation of this model and for a more realistic model of non-random abscission, which could improve our understanding of wind-driven spread of dandelions, bringing us a crucial step closer to understanding dandelion’s ecological success.

This directional bias mechanism could have evolved due to decreased fitness if offspring land near the parent plant. Having this asymmetric abscission response not only lessens abscission probability in downwinds, but also against the constant force of gravity, which is pulling the seeds towards the ground. The plants have evolved with the constant stimulus of gravity, making it plausible that the increased tissue region in the negative direction came about to buffer against the torque caused by gravity (and downwinds) acting on the seeds.

The observed directional bias of seed abscission most likely shapes the spatial profile of seed dispersal. Plants that exhibit directional abscission should have seeds that are more likely to disperse in different directions, leading to a larger spread of propagules. This would not only decrease the density of seeds landing in the same area, limiting competition, but also allow the propagules to potentially reach areas with better environmental conditions for plant maturation, being advantageous for higher offspring survival. Other plants in the Asteraceae family with anemochorous seed dispersal may also have similar structures that lead to directional sensitivity, e.g. daisies, asters and lettuce. These findings highlight the effect of wind direction on seed abscission and need to be considered for dispersal models to accurately predict the population dynamics and dispersal kernels of wind-dispersed plants.

## Data Availability

All data and code used in the analysis are available in eCommons [32]. Supplementary material is available online [33].
